# Highly Permeable Sulfonated Graphene-Based Composite Membranes for Electrochemically Enhanced Nanofiltration

**DOI:** 10.3390/polym14153068

**Published:** 2022-07-29

**Authors:** Junjie Wang, Mingyu Li, Gaoliang Wei

**Affiliations:** 1 Key Laboratory of Industrial Ecology and Environmental Engineering (Ministry of Education, China), School of Environmental Science and Technology, Dalian University of Technology, Dalian 116024, China; wangjj9987@163.com; 2 Key Laboratory of Groundwater Resources and Environment (Ministry of Education), College of New Energy and Environment, Jilin University, Changchun 130021, China; mingyuli@jlu.edu.cn

**Keywords:** composite membrane, porous materials, sulfonated graphene, nanofiltration, electrochemistry

## Abstract

A sulfophenyl-functionalized reduced graphene oxide (SrGO) membrane is prepared. The SrGO membranes have a high charge density in water and could provide many atomically smooth nanochannels, because of their strong ionized-SO_3_H groups and low oxygen content. Therefore, the SrGO membranes have an excellent performance in terms of high permeance and high rejection ability. The permeance of SrGO membranes could be up to 118.2 L m^−2^ h^−1^ bar^−1^, which is 7.6 times higher than that of GO membrane (15.5 L m^−2^ h^−1^ bar^−1^). Benefiting from their good electrical conductivity, the SrGO membranes could also function as an electrode and demonstrate a significantly increased rejection toward negatively charged molecules and positively charged heavy metal ions such as Cu^2+^, Cr^3+^ and Cd^2+^, if given an appropriate negative potential. The rejection ratios of these metal ions can be increased from <20% at 0 V to >99% at 2.0 V. This is attributed to the enhanced electrostatic repulsion between the SrGO membrane and the like-charged molecules, and the increased electrostatic adsorption and electrochemical reduction in these heavy metal ions on the membranes. This study is expected to contribute to efficient water treatment and the advance of graphene-based membranes.

## 1. Introduction

Membranes have found many applications in the fields of water purification and wastewater treatment because of their many advantages, such as small carbon footprint, high separation efficiency, and easy operation [1,2,3,4]. The membranes should be theoretically ultra-highly permeable, and, simultaneously, have an ultrahigh selectivity. However, traditional membranes typically suffer from a trade-off between their permeance and selectivity, because of their thick separation layer, wide pore size distribution, as well as rough and irregular pore channels. Therefore, it is of great significance to develop next-generation membranes with both high permeability and high selectivity for efficient water treatment.

Nanotechnology and nanomaterials have gained lots of interest in many fields [5,6,7], their advances may provide solutions to the problem. For example, laminar graphene oxide (GO) membranes have attracted much interest in nanofiltration because of their potential excellent permeability and selectivity [8,9,10,11]. GO nanosheets have atomically smooth non-oxidized regions, and can construct particular 2D nanochannels for ultrafast water transport [8]. GO nanosheets also have oxidized regions decorated with many oxygen groups including carboxyl (-COOH), epoxy (-O-) and hydroxyl (-OH) groups [12], which could hinder the water transport by the formation of hydrogen bonds [12]. Therefore, thermal or chemical reduction in the GO membranes could theoretically increase their permeance because of an increase in non-oxidized regions and a decrease in oxygen groups, which has been evidenced by molecular dynamics simulations [13,14]. However, many previous studies have shown the converse results—the permeance of the GO membranes usually decreases after reduction [15,16,17]. The principal reason is found to be the significant decrease (even to <0.4 nm) of their interlayer spacing. Such a narrow spacing cannot accommodate a layer of water molecules [18]. In addition, reduction-induced decrease in -COOH would reduce charge density on the surface of the membrane and then weaken the electrostatic repulsion between membrane surface and negatively charged ions or molecules. This may lower their rejection ability, since the electrostatic repulsion contributes largely to the rejection of charged species.

It is inferred that surface modification of the reduced graphene oxide, for example, substitution of -COOH groups by strongly ionized ones, may avoid such problems. The strongly ionized groups could increase charge density of the membrane, since many -COOH groups (weakly ionized groups) on membranes may not be ionized in acidic solutions. Additionally, the highly charged groups could increase electrostatic repulsion between graphene nanosheets in water, and prevent them from restacking into graphite structures. Because of the partial removal of oxygen groups after reduction, the surface-modified graphene could be highly conductive. The charge density of resultant graphene membranes could be further increased by giving them a negative potential [19,20], and, therefore, their rejection ability toward negatively charged ions and molecules could also be increased. At the same time, the negative potential could also induce the chemical reduction in some metal ions such as Cu^2+^, Cr^3+^ and Cd^2+^, resulting in their efficient removal from water.

In this study, we report a novel electrically conductive, sulfophenyl-functionalized, reduced GO (SrGO)-based composite membrane for nanofiltration. The -SO_3_H groups could be entirely ionized in water and are expected to endow a high charge density. In addition, the SrGO membranes have less epoxy and hydroxyl groups, and could possess more atomically smooth nanochannels and a higher permeance than GO membranes. In consideration of their good electrical conductivity, the SrGO membranes are also expected to show improved performance under electrochemical assistance.

## 2. Materials and Methods

### 2.1. Materials

Pristine multi-walled carbon nanotubes (CNTs, 20–40 nm in diameter, 10–30 μm in length) were obtained from Tanfeng Graphene Technology Co., Ltd. (Suzhou, China). Polyacrylonitrile (PAN) ultrafiltration membranes (average pore size of 10 nm) were purchased from Guangzhou Haitao Water Purification Equipment Co., Ltd. (Guangzhou, China). Graphite powders (5000 mesh) and other chemicals were all purchased from Aladdin company (Shanghai, China). The water used in experiments was an ultrapure water with a resistance of >18 MΩ. The physical and chemical properties of the materials used were summarized in Table S1.

### 2.2. Preparation of SrGO-Based Composite Membranes

**Figure 1:**
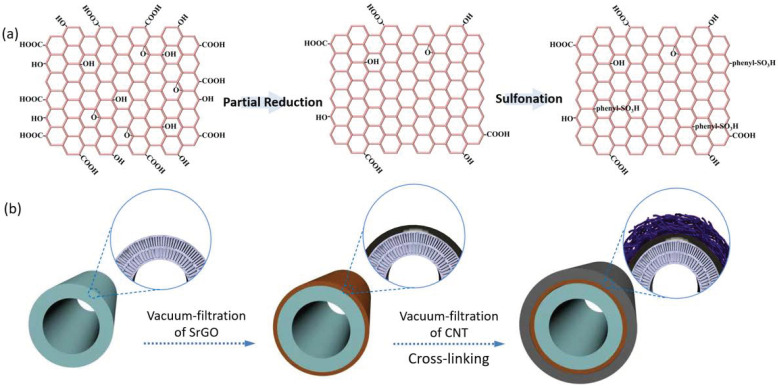
(**a**) Schematic illustration for the structures of GO, partially reduced GO and SrGO; (**b**) Schematic illustration for the preparation of SrGO membranes.

To prevent the SrGO separation layer from falling off substrates, we designed an asymmetrical hollow fiber membrane with the SrGO separation layer sandwiched between a porous CNT layer and substrate. The CNT dispersion was prepared according to a previously reported method [21]. The SrGO nanosheets (schematically shown in Figure 1a) were fabricated via the Si’s method [22]. To fabricate the membrane, a polyacrylonitrile (PAN) hollow fiber membrane was vertically inserted into a SrGO dispersion (50 mg L^−1^) after its lower end was sealed with epoxy resin, and then the other end was connected with a vacuum pump. The SrGO nanosheets were filtered onto the surface of the PAN membrane to form a thin layer, i.e., separation layer. Subsequently, the PAN membrane was removed from the SrGO dispersion and then inserted into a CNT dispersion (100 mg L^−1^). The CNTs would be deposited on the SrGO layer to form a sandwich structure (Figure 1b). The obtained composite membranes (abbreviated as SrGO membranes) were dried at ambient temperature (18–22 °C) for 24 h, followed by cross-linking with polyvinyl alcohol [21].

### 2.3. Characterization

The morphology and structure of sample was observed on a field emission scanning electron microscope (SEM, Hitachi S-4800, Japan). The samples were directly placed into SEM for observation without any special processing. The accelerating voltage was 10 KV, and the current was 5 μA. The atomic composition of sample was analyzed using an X-ray photoelectron spectrometer (XPS, Thermo ESCALAB 250, USA) using a 1486.6 eV Al Kα source. The interlayer spacing of the SrGO membrane was measured using a Bruker D8 ADVANC X-ray diffraction. Zeta potentials of the membranes were measured using a streaming current electrokinetic analyzer (SurPASS 3, Anton Paar GmbH, Austria). The thickness of SrGO nanosheets was measured by an atomic force microscopy (AFM, Bruker, Dimension Icon, Germany).

### 2.4. Performance Evaluation of SrGO Membranes

The membrane module used for electrochemically assisted filtration was schematically shown in Figure S1. The water permeance (*P*) of the SrGO membranes was calculated following the equation of *P* = *V*/(*St*Δ*p*), where *V* was the water volume (L) penetrating the membrane in a time interval (*t*, h), S was the effective membrane area (m^2^), and Δ*p* was the transmembrane pressure difference (bar).

Dye solutions (Evans blue (EB), Congo red (CR) or methyl blue (MB), 10 mg L^−1^), humic acid (HA, 10 mg L^−1^), salt solutions (CuSO_4_, Cr (NO_3_)_3_ or Cd (NO_3_)_2_, 1 mM), and dye/salt mixed solution (MB (10 mg L^−1^), CuSO_4_ (0.5 mM), Cr (NO_3_)_3_ (0.5 mM) and Cd (NO_3_)_2_ (0.5 mM)) were used to evaluate the separation performance of the SrGO membranes. To eliminate the contribution of adsorption to the rejection, the SrGO membranes were soaked in the solutions for 24 h before filtration. The concentrations of dyes and HA in filtrate were measured by an UV-visible spectrophotometer (Thermo Scientific Evolution 201), and the concentration of Cu^2+^, Cr^3+^ and Cd^2+^ was measured by an inductively coupled plasma mass spectrometry (Agilent 7850). The rejection ratio (*R*) was calculated following the equation of *R* = (*C*_0_ − *C*)/*C*_0_, where *C*_0_ and *C* were the concentrations of dyes, HA or metal ions in feed and filtrate, respectively.

## 3. Results

### 3.1. Characterization of SrGO Membranes

**Figure 2:**
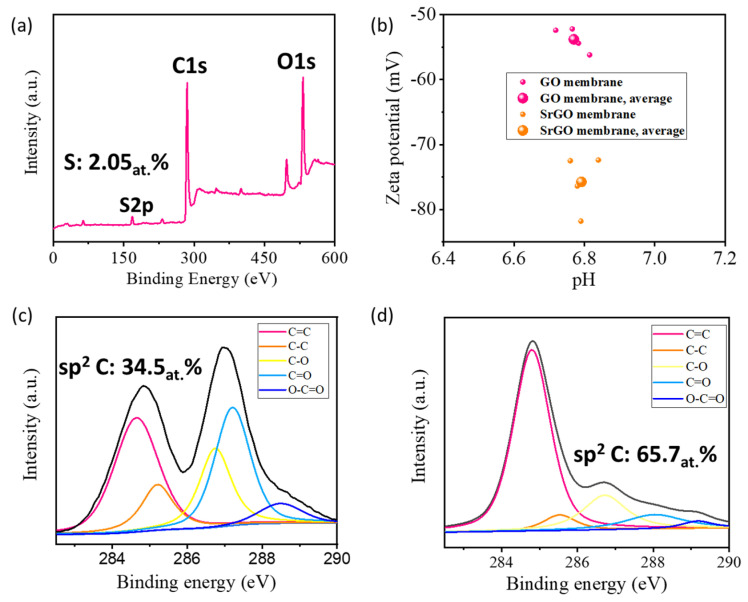
(**a**) XPS spectrum of a SrGO film; (**b**) Zeta potentials of a GO film and a SrGO film; (**c**) C1s spectrum of a GO film; (**d**) C1s spectrum of a SrGO film.

The thickness of the SrGO nanosheets is measured to be 1.25 nm by AFM (Figure S2), indicating they have a single- or double-layered structure. XPS spectrum of a SrGO film shows an obvious S2p characteristic peak, which suggests sulfophenyl groups have been decorated on reduced graphene oxide nanosheets with an S atom percentage of 2.05% (Figure 2a). As expected, the sulfophenyl groups can significantly increase the charge density of the membranes, as evidenced by the fact that the SrGO membranes have a larger negative zeta potential of −75.7 mV than GO membranes (−53.8 mV, Figure 2b). XPS C1s spectra show that the SrGO membranes have a sp^2^ C/sp^3^ C atomic ratio of 65.7%, which is much higher than that of GO membranes (34.5%, Figure 2c,d). This suggests that the SrGO membranes could potentially provide more atomically smooth nanochannels for water transport.

**Figure 3:**
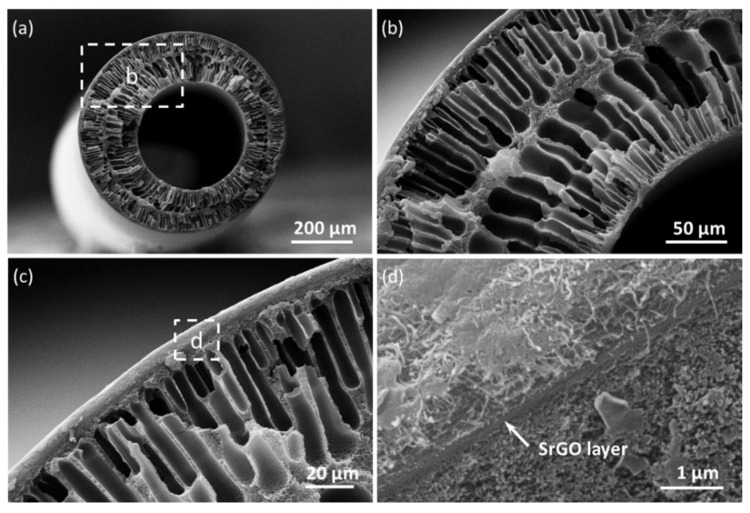
(**a**) Cross-sectional SEM images of the SrGO membranes; (**b**) Magnified area marked in **a**; (**c**) Magnified area marked in **b**; (**d**) Magnified area marked in **c**.

The SrGO membrane presents a typical hollow structure, with the SrGO separation layer sandwiched between the CNT layer and the PAN substrate (Figure 3a–c). The SrGO separation layer can be clearly observed from the SEM images (Figure 3d). The hollow structure can allow the SrGO layer to be omnidirectionally confined onto the substrate by the CNT layer, which would endow a highly stable structure that prevents the exfoliation of the hydrophilic SrGO layer [21].

### 3.2. Separation Performance of SrGO Membranes

**Figure 4:**
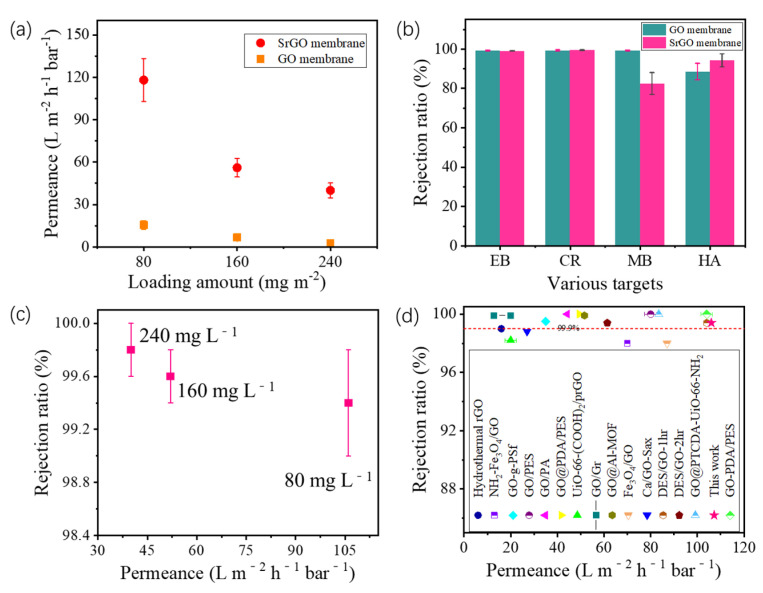
(**a**) Permeances of SrGO membranes and GO membranes with various loading amounts; (**b**) Rejection ratios of SrGO membranes and GO membranes toward various molecules; (**c**) Permeance and rejection ability toward CR of SrGO membranes with various loading amounts; (**d**) Performance comparison of the SrGO membranes and other graphene-based membranes in terms of their permeance and rejection ability toward CR (references are shown in Table S2).

Experimental results in Figure 4a show that the permeance of the SrGO membrane increases with a decrease in SrGO loading amount. For all SrGO membranes investigated, their permeances are much higher than those of GO membranes with same depositing amounts (Figure 4a). Specifically, at a depositing amount of 80 mg m^−2^, the SrGO membrane has a permeance of up to 118.2 L m^−2^ h^−1^ bar^−1^ (Figure 4a), which is 7.6 times higher than that of GO membrane (15.5 L m^−2^ h^−1^ bar^−1^).

Filtration results show that both the SrGO membrane and the GO membrane with a depositing amount of 80 mg m^−2^ have a rejection ratio of >99% toward EB and CR molecules (Figure 4b). However, during the filtration of HA molecules, the SrGO membranes have a higher rejection ratio than the GO membranes (86.2% vs. 78.5%). During filtration of the CR solution, the rejection ratios of SrGO membranes toward CR slightly decrease with the decrease in loading amount of SrGO nanosheets, but, are still higher than 99%. Specifically, the SrGO membranes with a SrGO loading amount of 80 mg L^−1^ have a rejection ratio of 99.4% at a permeance of 106 L m^−2^ h^−1^ bar^−1^ (Figure 4c). Their separation performance is higher than most of graphene-based membranes reported in other studies, in terms of permeance and rejection ability toward CR (Figure 4d and Table S2). The above results suggest that the high charge density and low oxygen content of the SrGO membranes could facilitate the water transport and molecule rejection.

**Figure 5:**
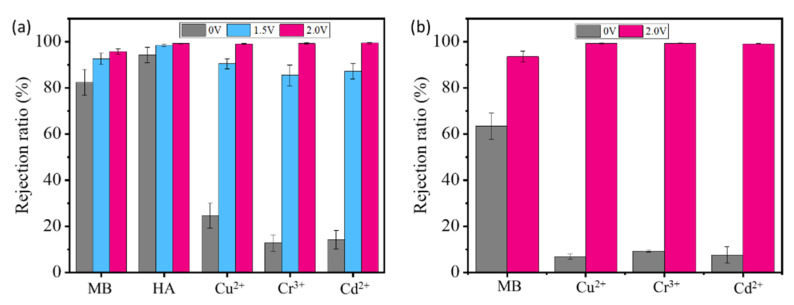
(**a**) Rejection ratios of various targets by SrGO membranes at different voltages during filtration of corresponding solutions; (**b**) Rejection ratios of various targets by SrGO membranes at 0 V and 2.0 V during filtration of their mixed solution.

Results show that both the SrGO membranes and the GO membranes have a low rejection toward Cu^2+^, Cr^3+^ and Cd^2+^ (Figure 5a), which could be largely attributed to swelling-induced expansion of their interlayer spacing. The interlayer spacing of the SrGO membranes is measured to be >1.76 nm (Figure S3); it is much larger than hydrated Cu^2+^, Cr^3+^ and Cd^2+^. The partial rejection of these ions should be attributed to the electrostatic and/or ion-π interactions between them and SrGO or GO. If a voltage of 1.5 V is applied on the filtration system with the SrGO membranes as cathode, their rejection ratios can be obviously increased from 82.4% to 92.5% toward MB, from 94.2% to 98.5% toward HA, from 24.6% to 90.4% toward Cu^2+^, from 12.8 to 85.4 for Cr^3+^ and from 14.2 to 87.2 for Cd^2+^. With an increase in the voltage to 2.0 V, their rejection ratios could be further increased (Figure 5a,b). Specifically, the metal ions are almost all rejected with a rejection ratio of >99% during filtration of their single-component solutions or mixed solution.

## 4. Discussion

The XPS results show that the SrGO membranes have a much higher sp^2^ C/sp^3^ C atomic ratio (Figure 2c,d), which is attributed to the removal of some oxygen-containing functional groups after chemical reduction. Because of the highly hydrophilic and charged -SO_3_H groups, the SrGO nanosheets do not restack completely after reduction, as evidenced by the weak peak at 2θ = 24.4° in XRD spectra (Figure S3). This suggests that the SrGO membranes could potentially provide more atomically smooth nanochannels for water transport than GO membranes or reduced GO membranes, which should be the main reason for the high permeance of SrGO membranes.

**Figure 6:**
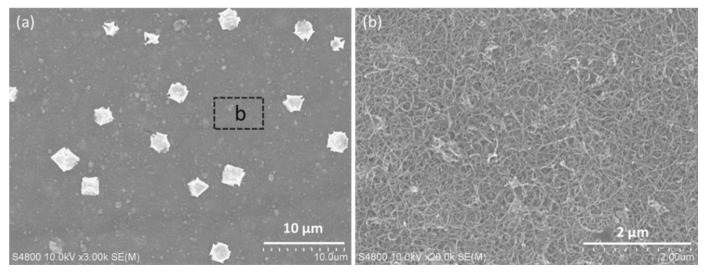
(**a**) SEM image of the surface of the SrGO membranes after filtration of copper nitrate solution under electrochemical assistance at 2.0 V; (**b**) High-resolution SEM image of the area marked in **a**.

Previous studies have revealed that the sieving rejection and electrostatic interactions are mainly responsible for the molecular and ionic separation of graphene-based laminar membranes [23,24]. The electrostatic repulsion between the membrane and charged molecules (or ions) could be further enhanced, if the membranes could additionally function as an electrode [25]. This is mainly attributed to the formation of electric double layer at the electrode/water interfacial, which is usually observed in supercapacitor [26,27]. Benefiting from the good electrical conductivity of SrGO (102 S m^−1^), the SrGO membranes are expected to achieve a higher performance under electrochemical assistance. During electrochemically assisted filtration, a negative potential can further increase the charge density of the SrGO membranes, and therefore enhance the electrostatic repulsion between membrane and negatively charged molecules/ions, which is the reason for the increased rejection ratios of MB and HA molecules. Additionally, a negative potential could promote electrostatic adsorption of heavy metal ions onto the SrGO membrane (including its CNT layer), and could also electrochemically reduce them into metal on CNT layer (Figure 6a and Figure S4), resulting in the high removal of the metal ions from water with rejection ratios of >99%. As they are electrochemically reduced before arriving at the SrGO separation layer (Figure 6a), the resulted metal particles could not block the interlayer channels of SrGO separation layer. SEM image shows that only a small proportion of the membrane area is occupied by the metal particles (Figure 6b); they do not decline the permeance of the SrGO membranes during electrochemically assisted filtration of these heavy metal ions, because of the interconnected pore channels and high flux of CNT layer. Additionally, the membranes could be facilely regenerated with a simple acid pickling process (Figure S4). The SrGO membranes are therefore advantageous over the vast majority of traditional membranes and other graphene-based membranes, in consideration of their high permeance and multifunction.

## 5. Conclusions

In conclusion, a novel sulfophenyl-functionalized reduced GO membrane was prepared. The SrGO membranes could have a much higher permeance (118.2 L m^−2^ h^−1^ bar^−1^) than GO membranes (15.5 L m^−2^ h^−1^ bar^−1^), when they have a similar rejection ability. This is mainly attributed to low oxygen content and strongly ionized -SO_3_H groups of SrGO membranes, as they could provide more water transport channels. If the SrGO membranes were given a negative potential, their rejection ratio toward negatively charged molecules such as MB and HA molecules could be obviously increased, and the rejection ratio toward heavy metal ions could be improved from <20% at 0 V to >99% at 2.0 V. This is benefited from their good electrical conductivity. The improved removal of the molecules should be attributed to the enhanced electrostatic repulsion between the SrGO membrane and the like-charged molecules, and the increased electrostatic adsorption and electrochemical reduction on the membranes account for the increased removal of these metal ions. This work is expected to provide new perspectives for future research on developing advanced membranes for efficient water treatment.

## Data Availability

Data is contained within the article and Supplementary Materials.

## Supplementary Materials

The following supporting information can be downloaded at: https://www.mdpi.com/article/10.3390/polym14153068/s1, Table S1: physical and chemical properties of the main materials used; Figure S1: scheme for the module used for electrochemically assisted filtration; Figure S2: atomic force microscope image of SrGO nanosheets; Figure S3: X-ray diffraction spectra of “dry” and wet SrGO films; Table S2: performance comparison of the SrGO membranes and other graphene-based membranes reported in the literature in terms of their permeances and rejection ability toward CR; Figure S4: SEM images of the surfaces of pristine SrGO membranes (**a**), after filtration of copper nitrate solution under electrochemical assistance at 2.0 V (**b**) and after soaking in 0.1 wt% HCl solution for 5 min (**c**). Refs. [28,29,30,31,32,33,34,35,36,37,38,39,40] are cited in Supplementary Materials file.
