# Supplementary material for: Highly Permeable Sulfonated Graphene-Based Composite Membranes for Electrochemically Enhanced Nanofiltration
Source: Polymers (Basel). 2022 Jul 29;14(15):3068. doi: 10.3390/polym14153068 (PMC9370331; doi:10.3390/polym14153068)
Supplement: Supplementary file 1 [file polymers-14-03068-s001.zip › polymers-1820337-supplementary.pdf]

Highly permeable sulfonated graphene-based composite membranes for electrochemically enhanced nanofiltration

Junjie Wang[a], Mingyu Li[b], Gaoliang Wei[a,b,*]

[a]Key Laboratory of Industrial Ecology and Environmental Engineering (Ministry of Education, China), School of Environmental Science and Technology, Dalian University of Technology, Dalian 116024, China

[b]Key Laboratory of Groundwater Resources and Environment (Ministry of Education), College of New Energy and Environment, Jilin University, Changchun 130021, China

Corresponding author: Gaoliang Wei

E-mail: glwei@dlut.edu.cn (G.L. Wei)

Table S1. Physical and chemical properties of the main materials used.

Materials	Chemical formula	Chemical/physical properties
CNT	-	20-40 nm in diameter, 10-30 μm in length, >95% in purification
PAN membrane	$(C_3H_3N)_n$	10 nm in average pore size, ~0.8 mm in outer diameter
Graphite powders	C	5000 mesh, >99% in purification
Potassium permanganate	$KMnO_4$	Analytical purity, oxidant
Concentrated sulfuric acid	H_2SO_4	~98wt.%, oxidant
Hydrogen peroxide	H_2O_2	~30wt.%
Sodium nitrite	$NaNO_2$	Analytical purity
4-aminobenzenesulfonic acid	$C_6H_7NO_3S$	Analytical purity
polyvinyl alcohol	$[C_2H_4O]_n$	~20000 Da in MW

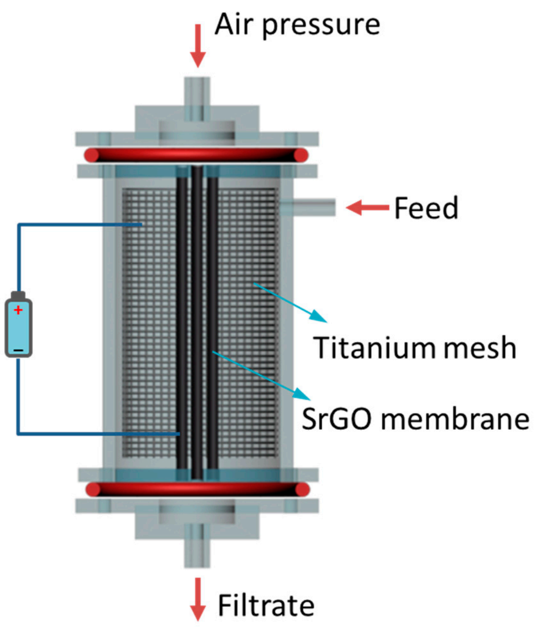

Figure S1. Scheme for the module used for electrochemically assisted filtration.

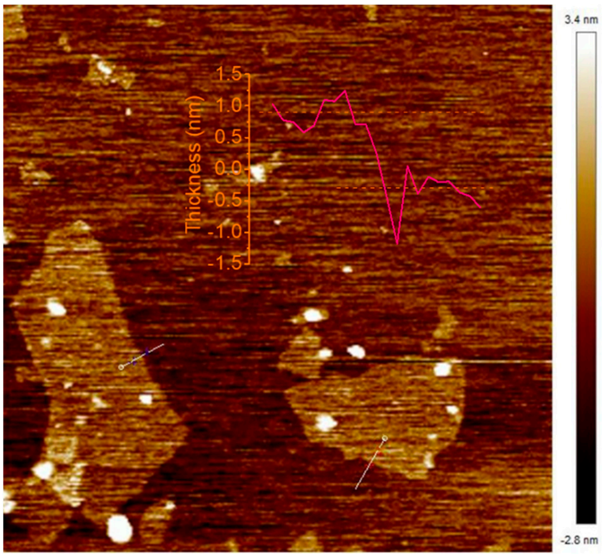

Figure S2. Atomic force microscope image of SrGO nanosheets.

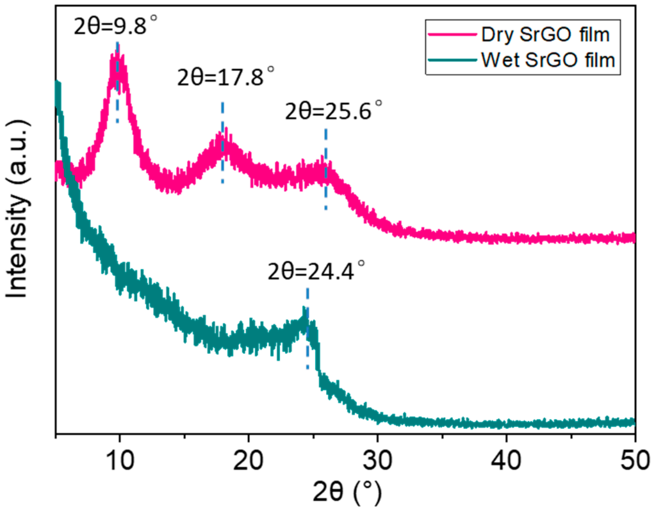

Figure S3. X-ray diffraction spectra of "dry" and wet SrGO films.

As shown in Figure S3, there is no characteristic peak in the spectrum of wet SrGO sample when 2θ is larger than 5°, which suggests the interlayer spacing is larger than 1.76 nm (calculated from Bragg equation, $2d\sin\theta = n\lambda$, where $n = 1$ and $\lambda = 1.54056$).

Table S2. Performance comparison of the SrGO membranes and other graphene-based membranes reported in literatures.

Samples	Permeance ($L\ m^{-2}\ h^{-1}\ bar^{-1}$)	Rejection (%)	References
Hydrothermal rGO membrane	~16	>99	[28]
NH_2-Fe_3O_4/GO membrane	~70	>98.0	[29]
GO-g-PSf membrane	~35	99.5	[30]
GO/PES	80 ± 2.5	100	[31]
GO-PDA/PES	104 ± 2.4	100	[31]
GO/PA membrane	44.2	100	[32]
GO@PDA/PES membrane	49.5	~100	[33]
UiO-66-$(COOH)_2$/prGO membrane	20.0 ± 2.5	98.2	[34]
GO/Gr membrane	12.8–20	99.9	[35]
GO@Al-MOF membrane	51.6	99.9	[36]
Fe_3O_4/GO membrane	87.01	98	[37]
Ca/GO-Sax membrane	27	>98.8	[38]
DES/GO-1hr membrane	104	99.4 ± 0.8	[39]
DES/GO-2hr membrane	61.4	>99.4	[39]
GO@PTCDA-UiO-66-NH_2 membrane	83.47	~100	[40]
SrGO membrane	106.0	99.4	This work

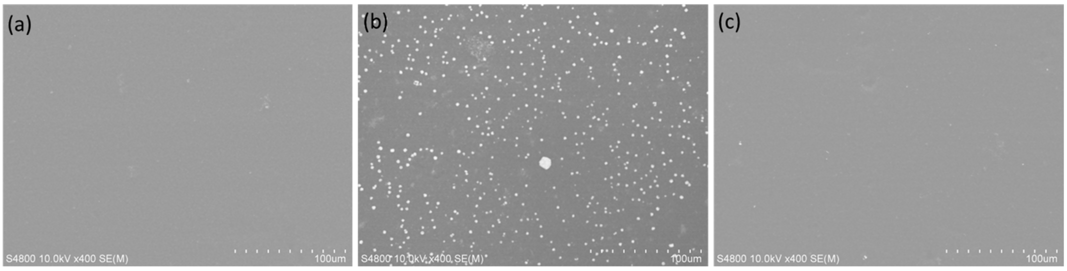

Figure S4. SEM images of the surface of pristine SrGO membranes (a), after filtration of copper nitrate solution under electrochemical assistance at 2.0 V (b) and after soaking in 0.1wt% HCl solution for 5 min (c).
